# Usability of the Gla-300 Injection Device Compared With Three Other Commercialized Disposable Insulin Pens: Results of an Interview-Based Survey

**DOI:** 10.1177/1932296815586219

**Published:** 2015-05-22

**Authors:** David Klonoff, Irina Nayberg, Frank Erbstein, Anna Cali, Claire Brulle-Wohlhueter, Thomas Haak

**Affiliations:** 1 Diabetes Research Institute, Mills-Peninsula Health Services, San Mateo, CA, USA; 2 Sanofi-Aventis Deutschland GmbH, Frankfurt am Main, Germany; 3 Sanofi, Paris, France; 4 Diabetes Klinik Bad Mergentheim, Bad Mergentheim, Germany

**Keywords:** Gla-300 SoloSTAR, insulin pen, interview-based survey, usability

New insulin glargine 300 U/mL (Gla-300; Toujeo^®^), recently approved for use in the United States and in Europe, has more stable and prolonged pharmacokinetic and pharmacodynamic profiles compared with glargine 100 U/mL (Gla-100; Lantus^®^).^1^ In clinical practice this translates into a comparable glucose-lowering effect and reduced risk of hypoglycemia.^2–4^ To deliver Gla-300, the well-known and widely used SoloSTAR^®^ pen has been adapted, allowing accurate delivery of insulin units in one-third of the volume compared with Gla-100.

To evaluate the perceptions of people with diabetes (users; 26 with type 1 and 228 with type 2 diabetes) and health care professionals with experience in prescribing insulin pens and training insulin pen users (trainers; n = 190) about the usability of the Gla-300 SoloSTAR, we conducted an interview-based survey in France, Germany, Spain, the United Kingdom, the United States, and Japan, comparing this device with three other commercialized disposable insulin pens: Gla-100 SoloSTAR (Sanofi, Paris, France), FlexPen^®^ (Novo Nordisk A/S Bagsværd, Denmark), and KwikPen™ (Eli Lilly & Co, Indianapolis, IN).

**Figure 1.**
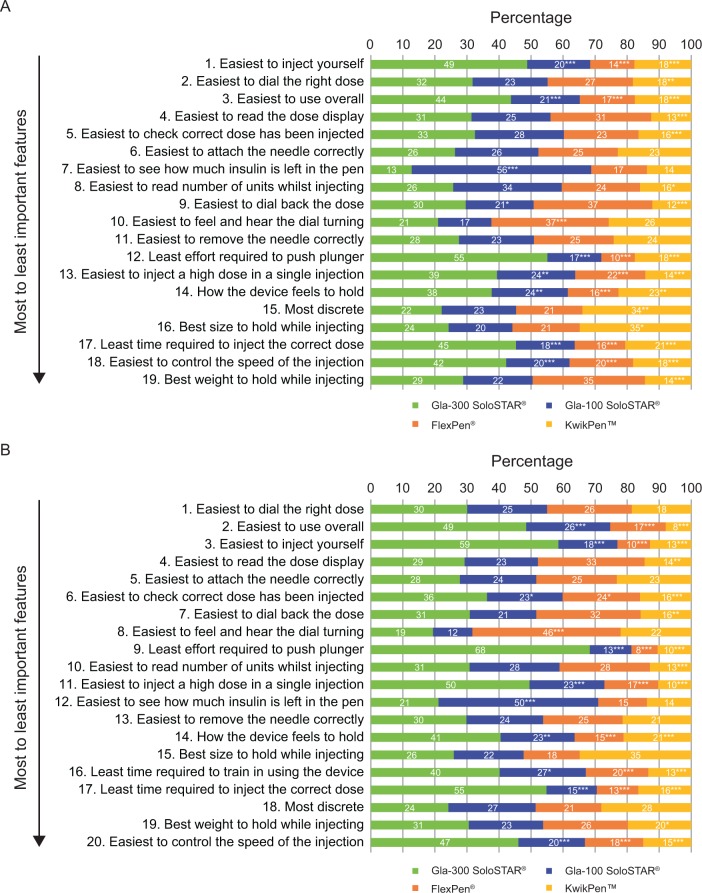
Percentage of users (n = 254) (A) and trainers (n = 190) (B) ranking each pen device in first place for each pen feature, in order of importance of pen features to the surveyed population (top is most important and bottom is least important). *P < .05 vs Gla-300 SoloSTAR. **P < .01 vs Gla-300 SoloSTAR. ***P < .001 vs Gla-300 SoloSTAR. Statistical significance estimated using two-tailed one-sample *t* test. Only trainers were asked to rank devices against the feature “Least time required to train in using the device.”

Each 75-minute face-to-face interview was conducted by an independent moderator. First, participants ranked a predefined list of features in order of importance to them, before the moderator demonstrated how to use each pen. Participants then tested each pen and ranked them (first, second, third or fourth) for each predefined feature (Figure 1). Only the identity of Gla-300 SoloSTAR was masked as the survey included pen-experienced users; to avoid bias caused by familiarity with other insulin pens, ≤16% of users were experienced with any one device. Participants were not informed of the survey sponsor.

Both users and trainers selected the same three features as being most important (Figure 1). Of these, users and trainers ranked Gla-300 SoloSTAR first for “Easiest to inject yourself” and “Easiest to use overall” significantly more often than other pens. In addition, the percentage of users and trainers ranking Gla-300 SoloSTAR in first place for “Easiest to dial the right dose” was numerically higher than for any other pen, although these differences did not always reach statistical significance.

Gla-300 SoloSTAR was also ranked first significantly more often than the other pens when considering the feature “Least effort required to push plunger” (Figure 1). This suggests that Gla-300 SoloSTAR may benefit people with reduced hand strength, although supporting data from a laboratory-based injection-force study would be of interest. Of note, Gla-100 SoloSTAR was perceived to perform significantly better than Gla-300 SoloSTAR for “Easiest to see how much insulin is left in the pen,” as was FlexPen for “Easiest to feel and hear the dial turning.”

This interview-based survey has limitations that should be considered when interpreting the results, including the use of only a single personal interview, the unmasked nature of the survey with regards to three of the tested pens, and the use of newly developed questionnaires that have not been validated or undergone psychometric testing. Further investigation of the use of Gla-300 SoloSTAR in clinical practice, ideally by insulin-naïve people with diabetes who may be reluctant to initiate insulin therapy, would therefore be of interest.

In conclusion, these survey results are promising because an insulin device that is easy to use and inject may contribute to increased adherence to insulin therapy,^5^ and therefore improve glycemic management.
